# Neuron Replating, a Powerful and Versatile Approach to Study Early Aspects of Neuron Differentiation

**DOI:** 10.1523/ENEURO.0536-20.2021

**Published:** 2021-05-19

**Authors:** Felix Schneider, Thuy-An Duong, Marco B. Rust

**Affiliations:** 1 Molecular Neurobiology Group, Institute of Physiological Chemistry, University of Marburg, Marburg 35032, Germany; 2 Center for Mind, Brain and Behavior (CMBB), University of Marburg and Justus-Liebig-University Giessen, Marburg 35032, Germany; 3 DFG Research Training Group, Membrane Plasticity in Tissue Development and Remodeling, GRK 2213, University of Marburg, Marburg 35032, Germany

**Keywords:** actin, cofilin, growth, neurite, neuron replating, replating

## Abstract

Neuron differentiation includes formation and outgrowth of neurites that differentiate into axons or dendrites. Directed neurite outgrowth is controlled by growth cones that protrude and retract actin-rich structures to sense environmental cues. These cues control local actin filament dynamics, steer growth cones toward attractants and away from repellents, and navigate neurites through the developing brain. Rodent hippocampal neurons are widely used to study the mechanisms underlying neuron differentiation. Genetic manipulation of isolated neurons including gene inactivation or reporter gene expression can be achieved by classical transfections methods, but these methods are restricted to neurons cultured for several days, after neurite formation or outgrowth. Instead, electroporation allows gene manipulation before seeding. However, reporter gene expression usually takes up to 24 h, and time course of gene inactivation depends on the half live of the targeted mRNA and gene product. Hence, these methods do not allow to study early aspects of neuron differentiation. In the present study, we provide a detailed protocol in which we combined electroporation-based gene manipulation of mouse hippocampal neurons before initial seeding with a replating step after 2 d *in vitro* (DIV) that resets neurons into an undifferentiated stage. By categorizing neurons according to their differentiation stage, thorough morphometric analyses, live imaging of actin dynamics in growth cones as well as guidance cue-mediated growth cone morphologic changes, we demonstrate that differentiation and function of replated neurons did not differ from non-replated neurons. In summary, we provide a protocol that allows to thoroughly characterize differentiation of mouse primary hippocampal neurons.

## Significance Statement

Unraveling the molecular mechanisms that control neuron differentiation requires reporter gene expression or gene inactivation. In mouse primary hippocampal neurons, a widely used cellular system to study neuron differentiation, classical transfection methods are restricted to later stages of differentiation. Instead, electroporation allows genetic manipulation before seeding. However, time course of reporter gene expression or gene inactivation frequently hinders a full characterization of neuron differentiation, specifically of early stages. To circumvent this limitation, we combined electroporation-based genetic manipulation before initial seeding with a replating step after 2 d *in vitro* (DIV), which reset neurons into an undifferentiated stage. We show that replated neurons differentiated similar to non-replated neurons. We provide a detailed protocol that allows to comprehensively characterize the molecular mechanisms underlying neuron differentiation.

## Introduction

During differentiation, neurons undergo striking morphologic changes from spheres to polar cells possessing an axon and a highly branched dendritic compartment (Dotti et al., 1988; da Silva and Dotti, 2002). Essential steps during early neuron differentiation include the formation and outgrowth of neurites, which later differentiate into axons or dendrites. Directed neurite outgrowth depends on growth cones, structures at neurite tips enriched in actin filaments (F-actin) that steer neurites toward attractants and away from repellent cues and, hence, navigate neurites through the developing brain (Gomez and Letourneau, 2014). Cultured hippocampal neurons isolated from mice or rats are widely used cellular systems to study neuron differentiation as they readily polarize on a two-dimensional substrate at very low densities (Dotti et al., 1988; da Silva and Dotti, 2002). Genetic manipulation including gene silencing, gene deletion or reporter gene expression provide powerful approaches to study virtually all biological processes in cellular systems, including neuron differentiation. Electroporation-based nucleofection as well as classical transfection procedures such as liposome-based transfection or calcium phosphate precipitation are the most commonly applied methods for gene transfer into cultured hippocampal neurons as they are far less labor-intensive when compared with virus infection (Dudek et al., 2001; Ohki et al., 2001; Zeitelhofer et al., 2009; Viesselmann et al., 2011; Sun et al., 2013). Unfortunately, efficiency of classical transfection procedures is rather low and these approaches are convenient only for hippocampal neurons cultured for several days, e.g., at around 6 d *in vitro* (DIV) or later. Instead, nucleofection allows genetic manipulation of hippocampal neurons before seeding. However, expression of reporter genes usually takes up to 24 h, and more importantly, time course and efficiency of gene silencing or gene deletion depends on the half live of the targeted mRNA and gene product. Consequently, nucleofection of hippocampal neurons does not allow a thorough analysis of neuron differentiation, specifically not of early processes during neuron differentiation. Thus, experimental approaches are needed to circumvent these limitations. We here report a protocol to reset primary hippocampal neurons from embryonic mice at DIV2 into an undifferentiated stage. Before initial seeding, these neurons can be manipulated genetically by means of nucleofection. We show that a combination of nucleofection and replating allows to study early aspects of neuron differentiation.

## Materials and Methods

### Mice

Generation of ADF−/−/Cfl1^flx/flx^ mice has been reported before (Bellenchi et al., 2007; Wolf et al., 2015; Zimmermann et al., 2015). Mice were housed with food and water available *ad libitum* on 12/12 h light/dark cycles. Treatment of mice was in accordance with the German law for conducting animal experiments and followed the guidelines for the care and use of laboratory animals of the National Institutes of Health. Killing of mice has been approved by internal animal welfare authorities (references: AK-5-2014, AK-6-2014, AK-12-2020). Genetic inactivation of Cfl1 in neurons from ADF−/−/Cfl1^flx/flx^ mice was achieved by nucleofection of catalytic active mCherry-Cre. ADF−/−/Cfl1^flx/flx^ neurons expressing a mutant, catalytic inactive mCherry-Cre served as controls. Both constructs have been achieved from the Solecki lab (Kullmann et al., 2020).

### Hippocampus dissection and neuron isolation

One day before neuron isolation, glass cover slips (13 mm in diameter, VWR) were placed into 24-well plates and coated overnight with 0.1 mg/ml poly-L-lysine hydrobromid (dilution of 1 mg/ml poly-L-lysine with 0.1 m boric acid at pH 8.5) in a humidified incubator at 37°C and 5% CO_2_. For replating, 24-well plates without cover slips were coated with 0.05 mg/ml poly-L-lysine hydrobromid and similar incubated as above. On the day of neuron isolation, plates were washed twice with ddH_2_O and equilibrated either with 500-μl nucleofection medium (DMEM-31966; Invitrogen) supplemented with 10% fetal bovine serum (FBS; Invitrogen) or for non-nucleofected neurons with neurobasal (NB; Invitrogen) medium. Mice of either sex were killed at embryonic day (E)18.5 by decapitation, and brains were dissected on ice in Leibovitz’s L15-Medium with 7 mm HEPES (L15+H, Invitrogen). After removal of the meninges, hippocampi of each embryo were isolated and collected in a tube containing cooled L15+H. Thereafter, medium was replaced by 500-μl prewarmed TrypLE Express (Invitrogen) per embryo and incubated for 6 min at 37°C. Subsequently, hippocampi were washed twice with NB medium containing 2% B27, 2 mm GlutaMax, 100 μg/ml streptomycin, and 100 U/ml penicillin (NB+, Invitrogen). After washing, neurons were triturated in 1 ml NB+ by pipetting seven times up and down with a P1000 pipette. Neuron solution was filled up to 1 ml NB+ medium per embryo and density was calculated by using a hemocytometer. Thereafter, neurons were plated at a density of 60,000 cells per well. 5 h after plating, medium was completely replaced by NB+ medium.

### Electroporation of hippocampal neurons

In some experiments, neurons were electroporated before plating. In these experiments, electroporation was performed according to manufacturer’s protocol by using the Amaxa P3 Primary Cell 4D-Nucleofector X kit L (Lonza) and 4D-Nucleofector (Lonza). For nucleofection, 250,000 neurons were transfected with 3-μg plasmid and the entire neuron suspension was plated in a single well of a 24-well plate in nucleofection medium; 5 h after plating, medium was completely replaced by NB+ medium.

### Replating of hippocampal neurons

At DIV2, neurons were detached and plated again (replated) on cover slips. Before replating, coverslips were prepared as described above. For replating, condition medium (350-μl medium from each well + 200 μl fresh NB+ medium for each well) was collected and kept in the water bath at 37°C. Remaining medium was aspirated, replaced with prewarmed 500-μl TrypLE Express per well and incubated for 15 min in the humidified incubator. To detach the cells after incubation, the bottom of the well was rinsed twice with the TrypLE Express, and 500-μl prewarmed NB+ medium was added to stop enzymatic reaction. Again, the bottom of the well was rinsed twice with the medium-enzyme solution and then completely transferred in to 1.5-ml cups and centrifuged for 5 min with 7000 rpm. Thereafter, pelleted neurons were re-suspended in 500-μl condition medium and plated on cover slips in 24-well plates and incubated at 37°% with 5% CO_2_ until further processing.

### Immunocytochemistry

One or 2 d after seeding or replating, neurons were fixed for 10 min in 4% paraformaldehyde in PBS under cytoskeleton preserving conditions (pH 7–7.5). After washing with PBS, neurons were incubated with 0.4% gelatin with 0.5% Triton X-100 in PBS (carrier solution) for 5 min, followed by incubation with the primary antibody rabbit anti-Dcx (1:500, Abcam; in carrier solution). After 90 min incubation, neurons were washed with PBS and incubated with Alexa Fluor 488-coupled phalloidin (1:100, ThermoFisher Scientific) to visualize F-actin and the secondary antibody anti-rabbit IgG coupled to Alexa Fluor 546 (1:500, Invitrogen; in carrier solution). After 60 min of incubation, neurons were washed with PBS and nuclei were stained with the DNA dye Hoechst (1:1000 in PBS, Invitrogen). Neurons were imaged with a Leica TCS SP5 II confocal microscope setup.

### Live cell imaging

For live cell imaging, neurons were seeded either directly after nucleofection or after replating in a poly-L-lysine hydrobromid-coated 22-mm glass-bottom dish and cultured for 1d. To measure actin turnover via fluorescence recovery after photobleaching (FRAP), neurons were transfected with GFP-actin (Robert Grosse lab) and imaged with a Leica TCS SP5 II in a chamber heated to 35°C. For imaging, neurons were washed once and then imaged in CO_2_-saturated HBS solution (Invitrogen), supplemented with 4.16 mm NaHCO_3_ and 2 mm CaCl_2_. For prebleaching condition, five images of growth cones were acquired and in total 65 images over a time course of 5 min during fluorescence recovery. Images were analyzed with ImageJ (Schindelin et al., 2012) and recovery curve and parameters were calculated with R. To assess retrograde F-actin flow of growth cones neurons were transfected with LifeAct-GFP (Robert Grosse lab) and imaged in a CO_2_-regulated chamber maintained at 37°C. Image acquisition was done with a Leica DMi8 Thunder microscope system and a Leica DFC9000 GTC camera, which acquired images every 5 s for 5 min. Kymograph generation and analysis was performed with ImageJ (Schindelin et al., 2012).

### Growth cone collapse assay and BDNF treatment

Neurons were treated for 60 min with 100 ng/ml BDNF (PeproTech), 1 μg/μl Ephrin A5 (R&D Systems) or 1 μg/μl Slit-1 (R&D Systems) before fixation. Images were acquired with a Leica TCS SP5 II microscope system and analyses were done with ImageJ (Schindelin et al., 2012). Growth cone size was measured for determining BDNF effects, whereas repellent cues treated growth cones were categorized into collapsed and non-collapsed according to previous studies (Müller et al., 1990).

### Statistics

Statistical tests were done in R or Sigma Plot. For comparing mean values between groups, Student’s *t* test or Mann–Whitney *U* test was performed. Analyzing the rescue conditions, ANOVA with *post hoc* test was used. Stage distribution and non-collapsed versus collapsed growth cones were tested for differences with χ^2^ test.

## Results

### Replating does not alter hippocampal neuron morphology

**Figure 1.**
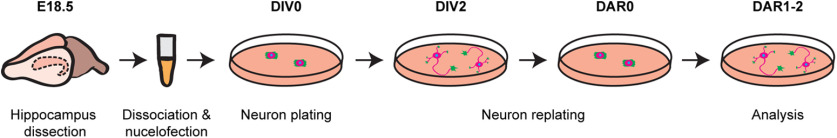
Scheme showing experimental procedure. Timeline and workflow of experimental approach including (1) isolation of hippocampal neurons from E18.5 mice; (2) nucleofection-based genetic manipulation before seeding that could be either reporter gene expression or gene inactivation; (3) culture of hippocampal neurons for 2 d; (4) replating of hippocampal neurons at DIV2 to reset them into an undifferentiated stage; (5) culture of replated neurons until further analyses.

**Figure 2.**
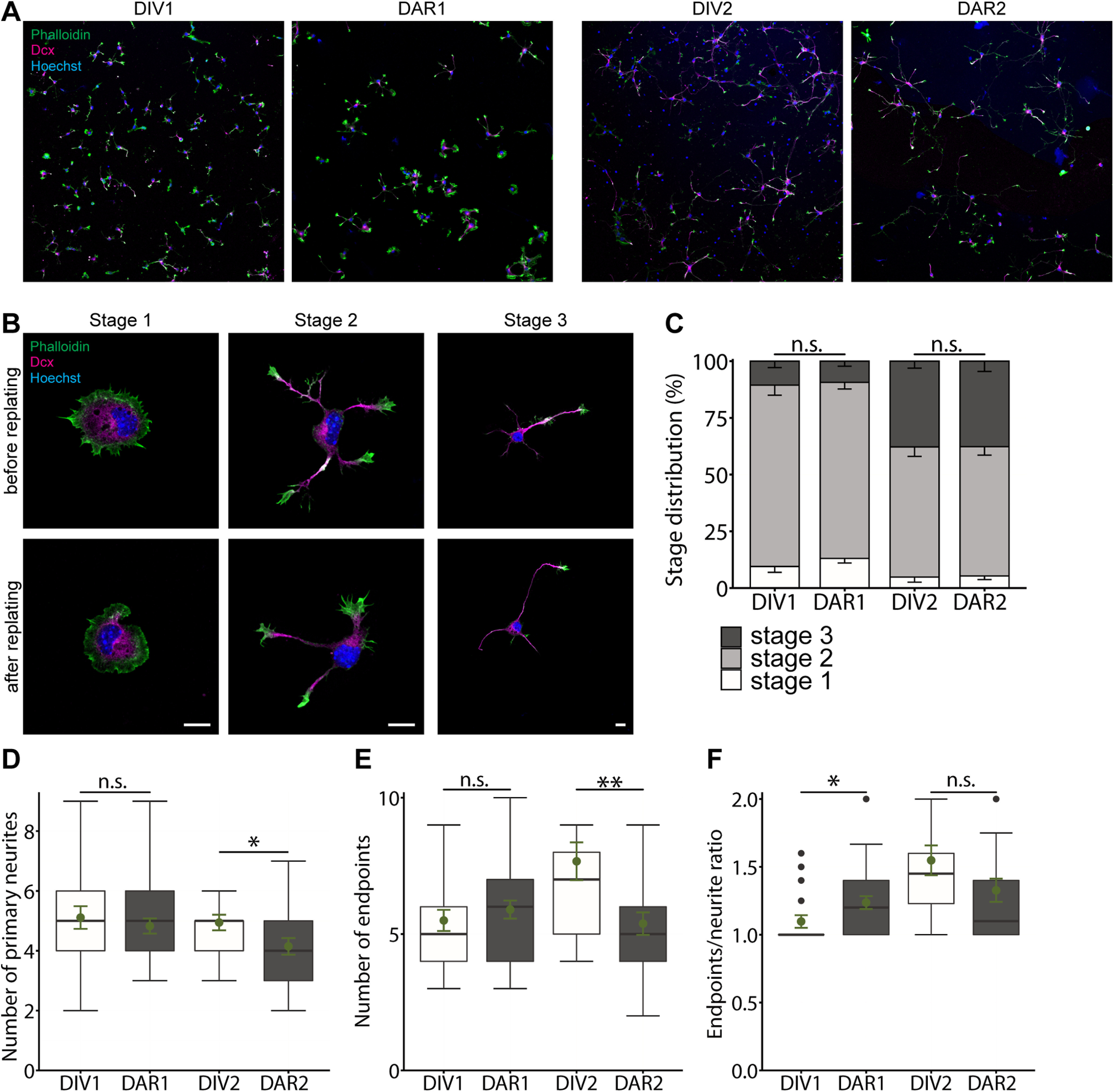
Replating neither alters differentiation nor gross morphology of hippocampal neurons. ***A***, Representative micrographs of mouse non-replated hippocampal neurons at DIV1 and DIV2 as well as replated neurons at DAR1 and DAR2. Neurons were stained with the F-actin marker phalloidin (green), with an antibody against Dcx (magenta) and the intercalating dye Hoechst (blue). ***B***, Representative micrographs of non-replated and replated stage 1, stage 2, and stage 3 neurons that have been used for morphometric analyses. ***C***, Stage distribution of non-replated and replated neurons. Graphs showing (***D***) numbers of primary neurites, (***E***) numbers of neurite endpoints as well as (***F***) primary neurite/neurite endpoint ratio in non-replated and replated neurons. Scale bars: 50 μm (***A***) and 10 μm (***B***); ns: *p* > 0.05, *p < 0.05, **p < 0.01. Green dots indicate mean values with SEM.

This study aimed at testing whether a combination of nucleofection and replating is a useful approach to study early aspects of hippocampal neuron differentiation. To do so, we isolated hippocampal neurons from C57Bl/6 mice at E18.5. Upon nucleofection, hippocampal neurons were seeded in 24-well plates and incubated at standard conditions (Fig. 1). After DIV2, we detached neurons by means of an enzymatic digest and mechanical treatment to reset them into an undifferentiated stage. Thereafter, hippocampal neurons were plated on cover slips and kept in culture, similar to non-replated neurons. To test whether this procedure affected neuron differentiation, we compared neurons 1 or 2 d after replating (DAR) with non-replated neurons at DIV1 or DIV2, respectively. We stained neurons with the F-actin marker phalloidin and an antibody against doublecortin (Dcx) that labeled neurites (Fig. 2*A*). This approach allowed us to categorize neurons according to their differentiation stage (Fig. 2*B*; Dotti et al., 1988). As expected, only a few non-replated DIV1 neurons remained in stage 1, i.e., they formed F-actin-enriched lamellipodia, but not yet neurites (Fig. 2*C*). The majority developed neurites, but not yet an axon and were assigned to stage 2, while a few neurons already possessed an axon and reached stage 3 (stage 1: 9.48 ± 2.55%; stage 2: 79.95 ± 4.43%, stage 3: 10.56 ± 2.83%, *n* > 180 cells from three independent experiments). Very similar to non-replated DIV1 neurons, we found a few neurons in stage 1 and stage 3 at DAR1, while the majority were assigned to stage 2 (stage 1: 13.05 ± 2.02%; stage 2: 77.59 ± 2.90%, stage 3: 9.36 ± 2.25%, *n* > 340/3). Comparison between DIV1 and DAR1 cultures revealed no difference in stage distribution (*p* = 0.44). At DIV2, the fraction of non-replated stage 3 neurons increased to roughly one third, and almost all other neurons were in stage 2 (stage 1: 4.81 ± 2.22%, stage 2: 57.39 ± 4.17%, stage 3: 37.80 ± 3.10%; *n* > 160/3). We found a similar stage distribution among DAR2 neurons (stage 1: 5.32 ± 1.59%, stage 2: 56.97 ± 3.71%, stage 3: 37.71 ± 4.56%; *n* > 240/3), with no difference when compared with DIV2 cultures (*p* = 0.81).

Antibody staining further allowed us to determine neuron morphology by counting the numbers of primary neurites and neurite endpoints and by calculating the ratio of primary neurites and neurite endpoints as a readout for neuron complexity. We determined these parameters in stage 2 neurons at DAR1 and DAR2 and compared them to non-replated neurons at DIV1 and DIV2, respectively. In DAR1 neurons, the numbers of primary neurites and neurite endpoints was not different from DIV1 neurons (neurites: DIV1: 5.11 ± 0.38, DAR1: 4.83 ± 0.25, *p* = 0.54; endpoints: DIV1: 5.50 ± 0.39, DAR1: 5.90 ± 0.33, *p* = 0.44; Fig. 2*D*,*E*). Instead, the neurite/endpoint ratio was slightly increased by roughly 10% in DAR1 neurons (DIV1: 1.10 ± 0.05, DAR1: 1.24 ± 0.05, *p* < 0.05; *n* > 20/3; Fig. 2*F*). Compared with DIV2 neurons, the neurite and endpoint numbers were slightly reduced by 8% and 30%, respectively, in DAR2 neurons (neurites: DIV2: 4.49 ± 0.26, DAR2: 4.12 ± 0.28, *p* < 0.05; endpoints: DIV2: 7.67 ± 0.70, DAR2: 5.38 ± 0.41, *p* < 0.01; *n* > 20/3; Fig. 2*D*,*E*). However, neuron complexity was similar to DIV2 neurons in DAR2 neurons (DIV2: 1.55 ± 0.11, DAR2: 1.33 ± 0.09, *p* = 0.12; Fig. 2*F*). Together, stage distribution did not differ between DAR1 and DIV1 cultures or between DAR2 and DIV2 cultures. Likewise, gross morphology of DAR1 and DAR2 neurons was similar to DIV1 and DIV2 neurons, respectively, and DAR2 neurons showed only minor changes in morphology.

### Replating does not alter growth cone size or morphology

**Figure 3.**
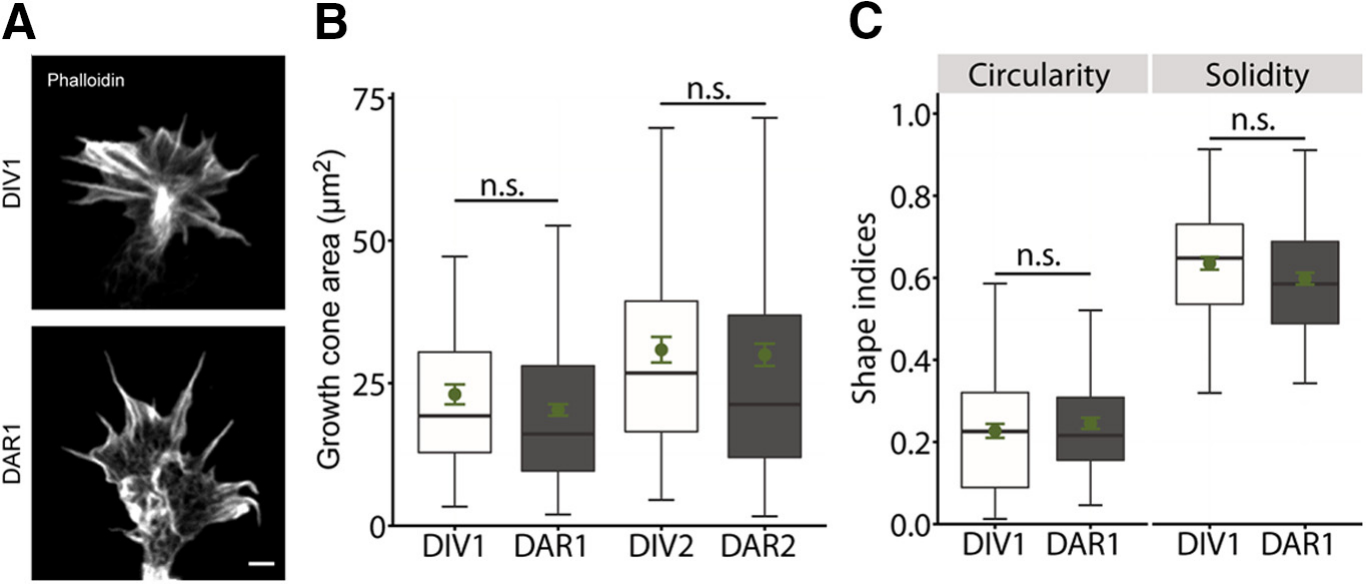
Replating does not alter growth cone size or morphology in hippocampal neurons. ***A***, Representative micrographs of phalloidin-labeled growth cones from non-replated and replated stage 2 neurons. ***B***, Growth cone size of non-replated and replated stage 2 neurons. ***C***, Growth cone morphology (solidity, circularity) of non-replated and replated stage 2 neurons. Scale bar: 2 μm (***A***); ns: *p* > 0.05. Green dots indicate mean values with SEM.

Next, we tested whether replating altered the morphology or function of growth cones, which are relevant for directed neurite outgrowth and neurite navigation through the developing brain. First, we exploited phalloidin-labeled neurons to determine growth cone size and morphology (Fig. 3*A*). For better comparison, we restricted this analysis to stage 2 neurons. In DIV1 and DIV2 neurons, growth cones size reached roughly 20 or 30 μm^2^, respectively (DIV1: 23.05 ± 1.74 μm^2^, *n* > 70/3; DIV2: 30.86 ± 2.25 μm^2^, *n* > 70/3; Fig. 3*B*). Growth cone size did not differ from non-replated DIV1 or DIV2 neurons in neurons from DAR1 or DAR2 cultures, respectively (DAR1: 20.33 ± 1.00 μm^2^, *n* > 100/3, *p* = 0.18; DAR2: 29.97 ± 1.95 μm^2^, *n* > 100/3, *p* = 0.76). Growth cone morphology was assessed by determining growth cone circularity (area divided by perimeter) and solidity (growth cone area divided by hull area), similar to previous studies (Chitsaz et al., 2015; Dos-Santos Carvalho et al., 2020). Both parameters were not different between growth cones from DAR1 and DIV1 neurons (solidity: DIV1: 0.63 ± 0.02, *n* > 70/3, DAR1: 0.60 ± 0.01, *n* > 90/3, *p* = 0.20; circularity: DIV1: 0.22 ± 0.02, *n* > 70/3, DAR1: 0.25 ± 0.01, *n* > 90/3, *p* = 0.33; Fig. 3*C*). Together, replating neither affected growth cone size nor morphology.

### Replating does not alter actin dynamics in growth cones

**Figure 4.**
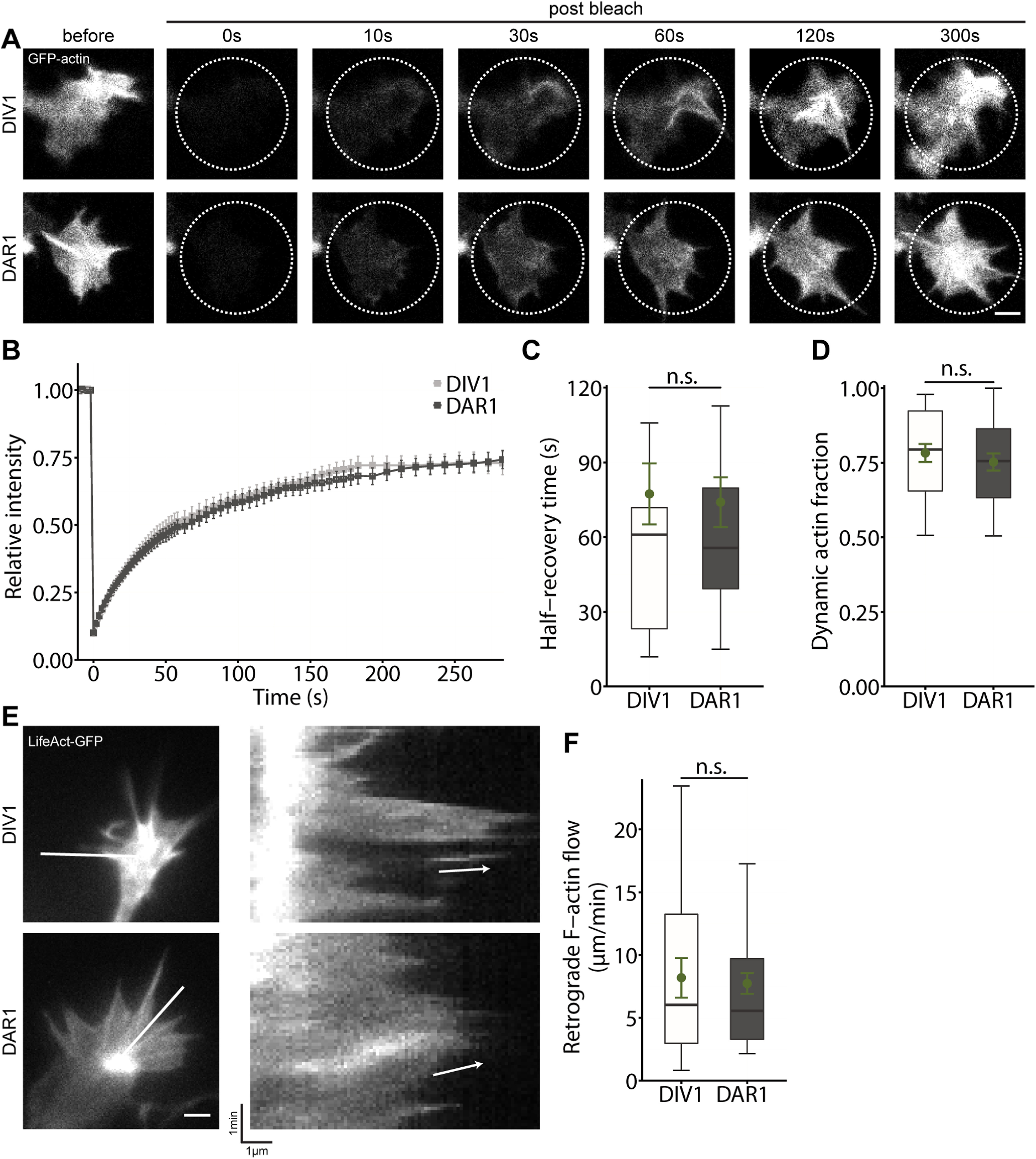
Replating does not impair actin dynamics in growth cones. ***A***, Image sequence of growth cones from GFP-actin-expressing non-replated and replated stage 2 neurons during FRAP analysis. ***B***, Recovery curves for GFP-actin in growth cones from stage 2 neurons at DIV1 and DAR1. ***C***, Half-recovery time of GFP-actin in growth cones during FRAP experiment. ***D***, Stable actin fraction in growth cones during FRAP experiments. ***E***, Representative micrographs of growth cones from LifeAct-GFP-expressing non-replated and replated neurons. Lines indicate where kymographs (shown on the right) have been generated from. Arrows indicate the retrograde F-actin flow. ***F***, Velocity of retrograde F-actin flow in growth cones. Scale bars: 2 μm (***A***, ***D***); ns: *p* > 0.05. Green dots indicate mean values with SEM.

Next, as functional readouts, we assessed actin dynamics in replated neurons. We electroporated neurons before seeding to express GFP-actin that allowed us to determine actin turnover in growth cones by FRAP, similar to previous studies (Flynn et al., 2012). We performed FRAP experiments in growth cones from DAR1 neurons and compared actin turnover to growth cones from non-replated DIV1 neurons. In growth cones from DIV1 neurons, GFP-actin rapidly recovered with a mean half-recovery time (t_½_) of 77.36 ± 12.29 s (*n* > 20/3; Fig. 4*A–C*; Movie 1). We noted a similar GFP-actin recovery in growth cones from DAR1 neurons, with no difference in t_½_ (74.04 ± 10.00 s, *n* > 20/3, *p* = 0.83; Fig. 4*A–C*; Movie 2). Further, we calculated the stable actin fraction that did not recover within the time frame of 300 s. This fraction was not different between growth cones from DIV1 and DAR1 neurons (DIV1: 0.78 ± 0.03, DAR1: 0.75 ± 0.03, *p* = 0.500; Fig. 4*D*). Additionally, we electroporated neurons before plating to express LifeAct-GFP, which allowed us to visualize F-actin in living neurons (Riedl et al., 2008; Flynn et al., 2012). F-actin appeared similarly dynamic in growth cones from DAR1 and DIV1 neurons (Movies 3, 4). Indeed, kymograph analysis revealed similar average retrograde flow velocity of F-actin in growth cones from both groups (DIV1: 8.18 ± 1.58 μm/min, *n* > 20/3, DAR1: 7.73 ± 0.82 μm/min, *n* > 50/3, *p* = 0.80; Fig. 4*E*,*F*). Together, replating neither affected actin turnover nor retrograde F-actin flow in growth cones.

Movie 1. Movie showing GFP-actin recovery upon bleaching in the growth cone of a non-replated neuron at DIV1. Upon bleaching fluorescence recovery was recorded over a time course of 3 min. Scale bar: 2 μm. 10.1523/ENEURO.0536-20.2021.video.1

Movie 2. Movie showing GFP-actin recovery upon bleaching in the growth cone of a replated neuron at DAR1. Upon bleaching fluorescence recovery was recorded over a time course of 3 min. Scale bar: 2 μm. 10.1523/ENEURO.0536-20.2021.video.2

Movie 3. Movie showing a growth cone from a LifeAct-GFP-transfected non-replated neuron at DIV1. Images were acquired every 5 s for 5 min. Scale bar: 2 μm. 10.1523/ENEURO.0536-20.2021.video.3

Movie 4. Movie showing a growth cone from a LifeAct-GFP-transfected replated neuron at DAR1. Images were acquired every 5 s for 5 min. Scale bar: 2 μm. 10.1523/ENEURO.0536-20.2021.video.4

### Growth cones from replated neurons respond normally to guidance cues

**Figure 5.**
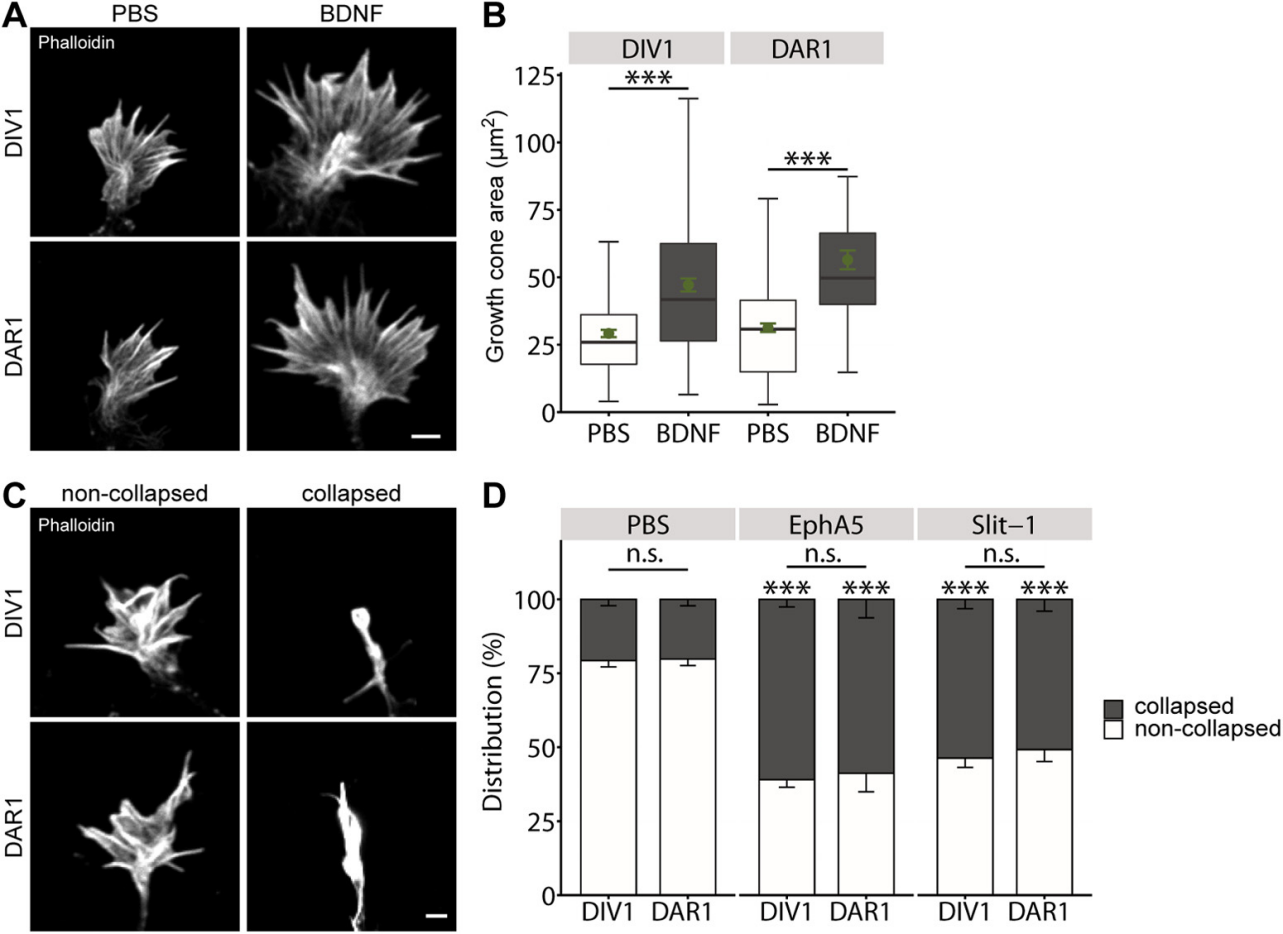
Normal response to guidance cues in growth cones from replated neurons. ***A***, Representative micrographs of phalloidin-stained growth cones from non-replated and replated neurons treated with either PBS or BDNF. ***B***, Growth cone size in non-replated and replated neurons treated with either PBS or BDNF. ***C***, Representative micrographs of phalloidin-stained collapsed and non-collapsed growth cones from non-replated and replated neurons. ***D***, Fractions of collapsed and non-collapsed growth cones in non-replated and replated neurons before and after treatment with EphA5 and Slit-1. Scale bars: 2 μm (***A***, ***C***); ns: *p* > 0.05, ***p < 0.001. Green dots in ***A*** indicate mean values with SEM.

Apart from studying actin dynamics, we tested whether growth cones from neurons of both groups respond differently to guidance cues. First, we determined growth cone size in phalloidin-stained DIV1 and DAR1 neurons on treatment with the neurotrophin brain-derived neurotrophic factor (BDNF). As expected (Meier et al., 2011), BDNF increased growth cone size in non-replated neurons by 62% when compared with PBS-treated controls (PBS: 29.17 ± 1.35 μm^2^, BDNF: 47.13 ± 2.40 μm^2^, *p* < 0.001, *n* > 130/3; Fig. 5*A*,*B*). BDNF similarly increased growth cone size in DAR1 neurons (PBS: 31.30 ± 1.59 μm^2^, BDNF: 56.45 ± 3.48 μm^2^, *p* < 0.001, *n* > 100/3). Hence, growth cones from DIV1 and DAR1 neurons respond similarly to BDNF.

Second, we investigated the effects of two different repellent cues, namely Ephrin A5 (EphA5) and Slit-1, on growth cones from non-replated and replated neurons (Meier et al., 2011; Ye et al., 2019). As a readout, we determined the fraction of collapsed growth cones in phalloidin-stained neurons on treatment with either EphA5 or Slit-1 and compared these fractions to PBS-treated control neurons (Fig. 5*C*). In agreement with normal growth cone morphology in replated neurons, the fraction of collapsed growth cones did not differ between DIV1 and DAR1 neurons before guidance cue treatment (DIV1: 20.71 ± 2.15%, DAR1: 20.20 ± 2.19%, *p* = 0.89, *n* > 200/3; Fig. 5*D*). EphA5 and Slit-1 increased the fraction of collapsed growth cones roughly threefold in DIV1 neurons (EphA5: 60.95 ± 2.59, *p* < 0.001, *n* > 300/3; Slit-1: 53.67 ± 3.17, *p* < 0.001, *n* > 300/3). Similarly, both repellent cues strongly increased the fraction of collapsed growth cones in DAR1 neurons (EphA5: 58.80 ± 6.26, *p* < 0.001, *n* > 210/3; Slit-1: 50.80 ± 4.04, *p* < 0.001, *n* > 200/3). Together, growth cones from non-replated and replated neurons respond similarly to the neurotrophin BDNF as well as the repellent cues EphA5 and Slit-1.

### Nucleofection-mediated gene inactivation allows to study early aspects of neuron differentiation in replated neurons

**Figure 6.**
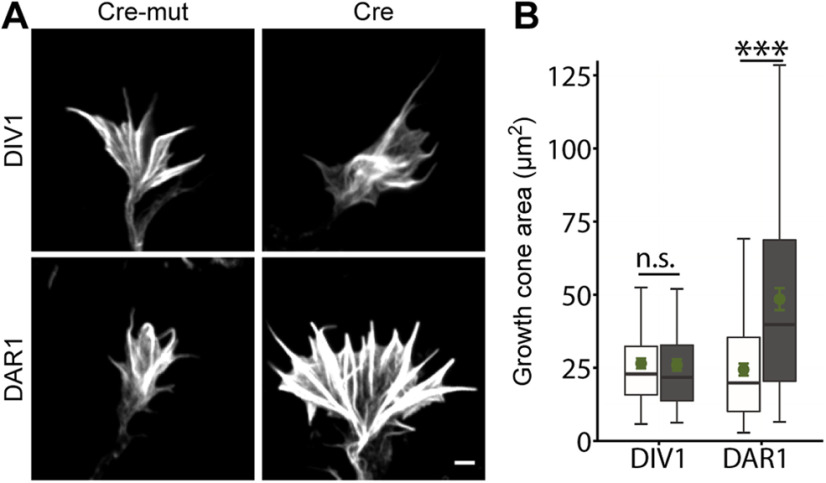
Replating allows studying the relevance of ADF/cofilin for early aspects of neuron differentiation. ***A***, Representative micrographs of phalloidin-stained growth cones from non-replated and replated ADF−/−/Cfl1^flx/flx^ neurons expressing either Cre or Cre-mut. ***B***, Growth cone size in non-replated and replated ADF−/−/Cfl1^flx/flx^ neurons expressing either Cre or Cre-mut. Scale bar: 2 μm (***A***); ns: *p* > 0.05, ***p < 0.001. Green dots indicate mean values with SEM.

The aforementioned approaches to test growth cone actin dynamics in replated neurons were based on nucleofection-based reporter gene expression. To extend our characterization of replated neurons to gene inactivation, we exploited primary hippocampal neurons from gene targeted mice (ADF−/−/Cfl1^flx/flx^) lacking the actin-binding protein ADF and additionally carrying a floxed allele of the ADF homolog cofilin1 (Bellenchi et al., 2007). We chose this mouse model for a proof of concept, because actin-depolymerizing proteins of the ADF/cofilin family have been previously implicated in growth cone morphology (Gomez and Letourneau, 2014; Omotade et al., 2017), and because previous studies revealed redundant functions of ADF and cofilin1 in neurons (Zimmermann et al., 2015; Wolf et al., 2015; Flynn et al., 2012). To inactivate cofilin1, we electroporated ADF−/−/Cfl1^flx/flx^ neurons before initial seeding with mCherry-tagged Cre recombinase (Cre), ADF−/−/Cfl1^flx/flx^ neurons expressing a catalytically inactive mCherry-Cre variant (Cre-mut) served as controls (Kullmann et al., 2020). We fixed Cre-expressing and Cre-mut-expressing ADF−/−/Cfl1^flx/flx^ neurons at either DIV1 or DAR1 and determined growth cone size on phalloidin staining (Fig. 6*A*). At DIV1, we found that growth cone size in Cre-expressing ADF−/−/Cfl1^flx/flx^ neurons was not different from Cre-mut-expressing controls (Cre-mut: 26.5 ± 1.72 μm^2^, Cre: 25.96 ± 1.95 μm^2^, *p* = 0.100, *n* > 30/3; Fig. 6*B*). Instead, growth cone size was strongly increased in Cre-expressing ADF−/−/Cfl1^flx/flx^ neurons at DAR1 when compared with Cre-mut-expressing controls (Cre-mut: 24.40 ± 2.2 μm^2^, Cre: 48.50 ± 3.74 μm^2^, *p* < 0.001, *n* > 80/3). Hence, ADF−/−/Cfl1^flx/flx^ neurons displayed the expected increase in growth cone size on genetic inactivation of ADF and cofilin1 at DAR1, but not at DIV1. Together, our replating protocol together with nucleofection-based gene inactivation before initial seeding allowed us to study the relevance of a gene of interest for early processes of neuron differentiation, thereby highlighting the utility of our approach.

## Discussion

In the present study we report a protocol to reset DIV2 primary mouse hippocampus neurons into an undifferentiated stage. We combined replating with nucleofection-based genetic manipulation (both reporter gene expression as well as gene inactivation by exploiting the Cre/loxP system) before initial seeding of primary neurons. This approach allows a thorough analysis of neuron differentiation including early processes such as neurite formation and outgrowth or growth cone function.

Replating of cultured neurons has been reported for various neuron subtypes including primary dorsal root ganglia (DRG) neurons, primary cortical neurons or stem cell (SC)-derived neurons (Caviedes et al., 1990a,b; Koechling et al., 2011; Saijilafu et al., 2013; Frey et al., 2015; Biswas and Kalil, 2018; Calabrese et al., 2019; Lee et al., 2020a). Neuron replating has been implemented to reduce neuron complexity and cell membrane surface area, thereby improving accessibility for electrophysiological recordings, because passive membrane properties such as membrane capacitance or resistance were altered (Caviedes et al., 1990a,b). Further, it has been implemented to transfer SC-derived neurons from normal cell culture dishes onto 384 wells before experiments (Calabrese et al., 2019), and it has been exploited as a paradigm of axon regeneration (Saijilafu et al., 2013; Frey et al., 2015; Lee et al., 2020a). These studies differed in the procedure applied, and some of them only included a brief and rather superficial description of the method. Moreover, these studies either did not focus on early aspects of neuron differentiation, did not systematically compare non-replated and replated neurons or did not combine replating with genetic manipulation. Hence, it remained unknown whether differentiation of replated neurons differed from non-replated neurons and whether a combination of genetic manipulation before initial seeding and replating allowed to study early aspects of neuron differentiation.

We compared cultured mouse hippocampal neurons that have been replated at DIV2 with non-replated neurons, focusing on early aspects of neuron differentiation up to 2 DAR. Our comparison included a categorization of neurons according to their differentiation stage as well as a thorough morphometric analysis. Neuron categorization did not reveal any differences between non-replated and replated neurons, thereby demonstrating that differentiation was largely preserved in replated neurons. Likewise, gross morphology was normal in replated neurons. However, they displayed some changes in neuron morphology, which are likely not biologically relevant. Our data demonstrated that our replating procedure successfully reset DIV2 primary hippocampal neurons into an undifferentiated stage and that replated neurons differentiated very similar to non-replated neurons. Hence, replated neurons faithfully reflect normal differentiation of hippocampal neurons.

Further, we combined our replating procedure with nucleofection-based transfection of hippocampal neurons before initial seeding. We expressed reporter genes such as GFP-actin or LifeAct-GFP that allowed us to determine actin turnover as well as F-actin dynamics in growth cones as functional readouts. By FRAP analysis, we found that actin turnover in growth cones was not different between replated and non-replated neurons. Similarly, retrograde F-actin flow was unchanged in replated neurons. These finding demonstrated that our replating procedure did not alter actin dynamics in growth cones and let us suggest normal growth cone functions in replated neurons. Indeed, growth cones from replated neurons did not differ to those from non-replated neurons in their response to the neurotrophin BDNF or the repellent cues EphA5 and Slit-1. Together, our analysis in hippocampal neurons did not reveal any gross defects in differentiation, morphology or growth cone function in hippocampal neurons induced by the replating procedure. In contrast to our findings, a recent study revealed functional differences between non-replated and replated DRG neurons. Specifically, this study showed that axon regeneration occurred in replated adult DRG neurons even when gene transcription was inhibited by blocking RNA Polymerase II, while axon formation and outgrowth in non-replated adult DRG neurons required RNA Polymerase II activity (Saijilafu et al., 2013). However, it remained unknown whether such functional differences between replated and non-replated neurons is restricted to a specific cell types, i.e., adult DRG neurons, or whether these differences are present in all CNS and PNS neurons.

Apart from nucleofection of reporter genes, we exploited the Cre/loxP system to genetically remove actin-depolymerizing proteins of the ADF/cofilin family that have been previously linked to growth cone morphology (Gomez and Letourneau, 2014; Omotade et al., 2017). While growth cone size was unchanged in non-replated Cre-expressing ADF−/−/Cfl1^flx/flx^ neurons at DIV1, it was strongly increased in replated Cre-expressing ADF−/−/Cfl1^flx/flx^ neurons at DAR1. Differences in growth cone size between Cre-expressing ADF−/−/Cfl1^flx/flx^ neurons at DIV1 and DAR1 can be easily explained by the fact that DAR1 neurons were 2 d longer in culture when compared with DIV1 neurons. Thus, DAR1 neurons had longer time to express Cre and to recombine the genome and, hence, to genetically remove cofilin1. In line with this, previous studies showed residual cofilin1 levels up to a few days on beginning of Cre expression in the mouse brain, but also in various cell types including isolated hippocampal neurons (Bellenchi et al., 2007; Rust et al., 2010; Flynn et al., 2012; Rehklau et al., 2012). Together, these data demonstrated that our replating protocol in combination with nucleofection-based gene inactivation allows us to study the relevance of a gene of interest for early aspects of neuron differentiation, different from nucleofected non-replated neurons. Hence, nucleofection combined with our replating protocol enables a more thorough analysis of neuron differentiation when compared with neurons that were nucleofected, but not replated.

In summary, we report a protocol to reset DIV2 primary mouse hippocampal neurons into an undifferentiated stage. This procedure is compatible with nucleofection-based genetic manipulation of primary neurons before their initial seeding. Our approach allowed us (1) to express fluorescent reporters during neuron differentiation that are needed to address specific biological processes such as actin dynamics in growth cones or (2) to inactivate a gene of interest to study its function in early aspects of neuron differentiation. This approach is highly flexible, straightforward and far less labor-intensive and expensive than previous approaches, (1) in which transgenic mice such as Lifeact-expressing strains were exploited to study actin dynamics during early differentiation in cultured hippocampal neurons (Flynn et al., 2012) or (2) which required the breeding and scarification of a large number of knock-out mice and their control littermates. Hence, our replating protocol is very helpful to reduce the number of experimental animals, and it thereby complies with the 3R principle for a more ethical use of animals in biomedical research (Russell and Burch, 1959; Lee et al., 2020b). While we here used expression of fluorescent reporters and Cre/loxP-based gene inactivation for a proof of principle, genetic manipulation can be easily expanded to gene silencing via RNA interference or other modes of gene deletion, e.g., by exploiting the CRISPR/Cas system. Taken together, a combination of nucleofection and replating of primary mouse hippocampal neurons is a powerful and versatile approach to comprehensively study the molecular mechanisms regulating neuron differentiation.

## Synthesis

Reviewing Editor: Francisca Bronfman, Universidad Andrés Bello Decisions are customarily a result of the Reviewing Editor and the peer reviewers coming together and discussing their recommendations until a consensus is reached. When revisions are invited, a fact-based synthesis statement explaining their decision and outlining what is needed to prepare a revision will be listed below.

The authors describe a method of replating neurons to reset the ‘undifferentiated’ state in order to enable studying early neuronal differentiation in vitro after genetic manipulation. Overall, the results are convincing and hold great potentials. However, while the paper adequately evaluated similarities in neurite outgrowth and stages of morphological differentiation between non-replated and replated neurons, it remains unclear whether molecular differences remain between the two conditions. This fact is overemphasized by the lack of novel implications. For example, since they are not able to observe differences in early differentiation properties in their replating neurons there is no indication of what additional information, they could achieve with that comparing to previous studies.

The manuscript will benefit if;

(i) They genetically manipulated some genes involved in early differentiation or function. These demonstrations will largely promote people to adapt to this protocol for their studies. Some examples are genes involved in polarity development.

(ii) Both types of neurons respond similarly to growth factors (or not)

Minor points to be addressed.

(i) Please confirm p-value reported on page 8 line 183 -- this is a trend (P=0.09), but the data is nearly identical.

(iii) Please fix p-value typo page 9 line 218.

(iv) Clarify; in Fig 1B, there seem to be distinct puncta of phalloidin staining surrounding the neuron soma in Stage 3 neurons before plating, which does not seem present in the after-plating image - is this representative of the results, and if so, what may be the cause for such a difference?

(v) Include in the discussion what is the capacity of this new protocol

(vi) Address in the discussion the paper by Saijilafu et al. (pmid: 24162165). They indicate that replating “... recapitulates biochemical and morphological properties of axon regeneration triggered by nerve injury...”, and as such, would imply transcriptional differences between DIV2 and DAR2 neurons.

(vii) The manuscript will benefit if the electrophysiological properties of replated neurons are discussed

## Author Response

Dear Dr. Bronfman.

Thank you very much for considering our manuscript ‘Neuron replating - a powerful

and versatile approach to study early aspects of neuron differentiation’ (#eN-OTM-0536-20) for publication in eNeuro and for allowing us to respond to the comments and

suggestions made by the reviewers.

The reviewers praised that our results are convincing and hold great potentials.

However, they phrased two major concerns that need to be addressed before publication

in eNeuro. Specifically, they asked i) to combine neuron replating with gene manipulation

and ii) to test whether replated neurons respond to growth factors similarly to non-replated neurons. In the revised manuscript, we addressed both concerns and include

additional data demonstrating i) that neuron replating can be successfully combined with

gene inactivation prior to initial seeding in order to study the relevance of a gene of

interest for early aspects of neuron differentiation (novel Fig. 6) and ii) that growth cones

from replated neurons respond to the neurotrophin BDNF or the repellent cues Ephrin A5

and Slit-1 similar to those from non-replated neurons (novel Fig. 5). In our point-to-point

reply below, we have highlighted the reviewers’ comments in bold and our detailed

response in regular font.

We hope that you and the reviewers will find the revised manuscript adequate for

publication in eNeuro.

In case of questions, please do not hesitate to contact me anytime.

On behalf of all co-authors, sincerely yours, the corresponding author.2

Manuscript Instructions

The sex of the species studied is not mentioned in the materials and methods

section. Please mention ‘male’, ‘female’, ‘x males and x females’, ‘of either sex’.

As pointed out in the second paragraph of the ‘Material and Methods’ section, we

sacrificed mice of either sex at embryonic day 18.5 for the isolation of primary

hippocampal neurons.

Major concerns

The manuscript will benefit if...

... they genetically manipulated some genes involved in early differentiation or

function. These demonstrations will largely promote people to adapt to this

protocol for their studies. Some examples are genes involved in polarity

development.

We thank the reviewers of this excellent suggestion. We agree that the utility of our

protocol will be much better promoted by combing our neuron replating protocol with

manipulation of genes that are known to be relevant for early aspects of neuron

differentiation. We therefore decided to inactivate actin-depolymerizing proteins of the

ADF/cofilin family prior to initial seeding, because ADF/cofilin has been earlier implicated

in the control of growth cone morphology (Gomez, J Neurochem 2014; Omotade, Mol

Cell Neurosci 2017). In the revised manuscript, we inactivated two ADF/cofilin family

members, namely ADF and cofilin1, by exploiting primary hippocampal neurons from

gene targeted mice that did not express ADF, but a floxed cofilin1 allele (ADF-/-

/Cfl1flx/flx).

Since ADF was shown to be dispensable for neuron differentiation and brain

development (Bellenchi, Genes Dev 2007), we did not expect any defects in neurons

from ADF-/-

/Cfl1flx/flx mice. However, cofilin1 was implicated in growth cone morphology

and neurons with compound inactivation of ADF and cofilin1 showed more severe

defects when compared to cofilin1 single knockout neurons (Wolf, Cereb Cortex 2015;

Zimmermann, Biol Psychiatry 2015; Flynn, Neuron 2012). To inactivate cofilin1 in

isolated ADF-/-

/Cfl1flx/flx neurons, we introduced by means of nucleofection mCherry-tagged Cre recombinase (Cre) prior to initial seeding, ADF-/-

/Cfl1flx/flx neurons expressing

a catalytic inactive mCherry-tagged Cre variant (Cre-mut) served as controls. We show

that growth cone size in Cre-expressing ADF-/-

/Cfl1flx/flx neurons was not different from

Cre-mut-expressing ADF-/-

/Cfl1flx/flx controls after one day in vitro (DIV1). Instead, growth

cones showed the expected size increase in Cre-expressing replated ADF-/-

/Cfl1flx/flx

neurons when compared to Cre-mut-expressing replated ADF-/-

/Cfl1flx/flx controls (novel 3

Fig. 6). Hence, when combined with nucleofection-based gene inactivation prior to initial

seeding, our replating protocol allows to thoroughly characterize the relevance of a gene

of interest for early aspects of neuron differentiation, thereby promoting the utility of our

protocol.

... both types of neurons respond similarly to growth factors (or not).

We thank the reviewers of this excellent suggestion. We agree that it is important to test

whether non-replated and replated neurons respond similarly to growth factors. To do so,

we decided to determine the effect of growth factors and other guidance cues on growth

cone size in neurons from both groups. As expected, brain-derived neurotrophic factor

(BDNF) enlarged growth cones in non-replated hippocampal neurons. In the revised

manuscript, we show that BDNF similarly enlarged growth cone size in replated neurons

(novel Fig. 5A-B). Moreover, we investigated the effects of the repellent cues Ephrin A5

(EphA5) and Slit-1 on growth cone size. Very similar to non-replated hippocampal

neurons, both EphA5 and Slit-1 increased the fractions of collapsed growth cones in

replated neurons roughly threefold (novel Fig. 5C-D). We concluded that non-replated

and replated respond similarly to guidance cues, thereby supporting the main

conclusions of our manuscript and further promoting the utility of our protocol.

Minor points

Please confirm p-value reported on page 8 line 183 - this is a trend (P=0.09), but

the data is nearly identical.

We thank the reviewers for indicating this. Indeed, this P value was not correct and we

like to apologize for the mistake. We corrected this mistake in the revised manuscript.

Please fix p-value typo page 9 line 218.

The typo is fixed in the revised manuscript.

Clarify; in Fig 1B, there seem to be distinct puncta of phalloidin staining

surrounding the neuron soma in Stage 3 neurons before plating, which does not

seem present in the after-plating image - is this representative of the results, and if

so, what may be the cause for such a difference?

We assume that Fig. 2B is meant here. We like to point out that these puncta are

staining artefacts that occurred both in replated and non-replated neurons.

Include in the discussion what is the capacity of this new protocol

We rephrased the discussion in the revised manuscript and we now better highlight the

capacity of our protocol. 4

Address in the discussion the paper by Saijilafu et al.

We thank the reviewers for mentioning the study by Saijilafu and colleagues (Saijilafu,

Nat Commun 2013), which we missed in our first manuscript. Interestingly, the authors

showed that replated and non-replated adult DRG neurons differed in their dependence

on RNA polymerase II. We included this study in the discussion of the revised

manuscript.

The manuscript will benefit if the electrophysiological properties of replated

neurons are discussed

We thank the reviewers for this suggestion. We agree, that a characterization of

electrophysiological properties is an additional possibility to characterize replated

neurons and to test whether a replating procedure affects neuron function. However, we

believe that our protocol is of higher interest for cell biologist studying neuron polarity,

neurite formation or neurite outgrowth, but less for electrophysiologists. Indeed,

electrophysiological properties of replated neurons have not been investigated in most

studies. Only a few studies included electrophysiological analysis and these studies

implemented a replating procedure of DRG neurons after 1 - 2 weeks in vitro and 24

hours before electrophysiological analyses to reduce neuron complexity and thereby

increase accessibility for path clamp recordings (Caviedes, J Neurosci Methods 1990).

Hence, only little is known about the electrophysiological properties of replated neurons

and a detailed discussion is therefore not possible. Nevertheless, we include some more

information about electrophysiological properties of replated neurons in the revised

manuscript.

References

Bellenchi GC, Gurniak CB, Perlas E, Middei S, Ammassari-Teule M, Witke W (2007) N-cofilin is associated with neuronal migration disorders and cell cycle control in the

cerebral cortex. Genes Dev 21:2347-57.

Caviedes P, Ault B, Rapoport SI (1990) Replating improves whole cell voltage clamp

recording of human fetal dorsal root ganglion neurons. J Neurosci Methods 35:57-61.

Flynn KC, Hellal F, Neukirchen D, Jacob S, Tahirovic S, Dupraz S, Stern S, Garvalov

BK, Gurniak C, Shaw AE, Meyn L, Wedlich-Söldner R, Bamburg JR, Small JV, Witke

W, Bradke F (2012). ADF/cofilin-mediated actin retrograde flow directs neurite

formation in the developing brain. Neuron 76:1091-107.5

Gomez TM, Letourneau PC (2014). Actin dynamics in growth cone motility and

navigation. J Neurochem. 129:221-234.

Omotade OF, Pollitt SL, Zheng JQ (2017). Actin-based growth cone motility and

guidance. Mol Cell Neurosci 84:4-10.

Saijilafu, Hur EM, Liu CM, Jiao Z, Xu WL, Zhou FQ (2013) PI3K-GSK3 signalling

regulates mammalian axon regeneration by inducing the expression of Smad1. Nat

Commun 4:2690.

Wolf M, Zimmermann AM, Görlich A, Gurniak CB, Sassoè-Pognetto M, Friauf E, Witke

W, Rust MB (2015) ADF/Cofilin Controls Synaptic Actin Dynamics and Regulates

Synaptic Vesicle Mobilization and Exocytosis. Cereb Cortex 25:2863-75.

Zimmermann AM, Jene T, Wolf M, Görlich A, Gurniak CB, Sassoè-Pognetto M, Witke W,

Friauf E, Rust MB (2015) Attention-Deficit/Hyperactivity Disorder-like Phenotype in a

Mouse Model with Impaired Actin Dynamics. Biol Psychiatry 78:95-106
